# Dietary Bioactive Diallyl Trisulfide in Cancer Prevention and Treatment

**DOI:** 10.3390/ijms18081645

**Published:** 2017-07-28

**Authors:** Michael T. Puccinelli, Silvia D. Stan

**Affiliations:** 1 Department of Nutrition Science, Purdue University, West Lafayette, IN 47907, USA; michaelpuccinelli@gmail.com; 2 Purdue University Center for Cancer Research, West Lafayette, IN 47907, USA

**Keywords:** diallyl trisulfide, *Allium*, cancer chemoprevention

## Abstract

Bioactive dietary agents have been shown to regulate multiple cancer hallmark pathways. Epidemiologic studies have linked consumption of *Allium* vegetables, such as garlic and onions, to decreased incidence of cancer. Diallyl trisulfide (DATS), a bioactive compound derived from *Allium* vegetables, has been investigated as an anti-cancer and chemopreventive agent. Preclinical studies provide ample evidence that DATS regulates multiple cancer hallmark pathways including cell cycle, apoptosis, angiogenesis, invasion, and metastasis. DATS has been shown to arrest cancer cells at multiple stages of the cell cycle with the G2/M arrest being the most widely reported. Additionally, increased pro-apoptotic capacity as a result of regulating intrinsic and extrinsic apoptotic pathway components has been widely reported following DATS treatment. Invasion, migration, and angiogenesis represent emerging targets of DATS and support its anti-cancer properties. This review summarizes DATS mechanisms of action as an anti-cancer and chemopreventive agent. These studies provide rationale for future investigation into its use as a cancer chemopreventive agent.

## 1. Introduction

Cancer is a major public health problem in every region of the world, with a projected incidence of 22.2 million of cancer cases by 2030 [1]. In 2017, an estimated 1.68 million people will be diagnosed with cancer leading to 600,920 deaths in the United States alone [2]. For centuries, whole foods including fruits, vegetables, and spices have been used to prevent and treat a variety of ailments such as wounds, inflammation, and infection. More recently, bioactive agents derived from these whole foods have been shown to display anti-microbial, anti-inflammatory, antioxidant, and anti-cancer effects.

Plants of the *Allium* genus, such as garlic and onions, have long been known to have medicinal qualities [3,4]. Research has determined that organosulfur compounds (OSCs) are the main bioactive agents responsible for the observed beneficial effects. Diallyl trisulfide (DATS), a bioactive OSC found in garlic, is reported to modulate disease states such as cancer, infection, and metabolic syndrome [5]. This review summarizes findings of DATS mechanisms of action relevant for cancer biology and discusses its use as an anti-cancer and chemopreventive agent.

## 2. Epidemiological Studies

Intake of *Allium* vegetables has been associated with reduced risk of various cancer types [6,7,8,9,10,11,12,13,14,15,16]. Multiple meta-analyses and epidemiological studies correlate *Allium* vegetable or garlic intake with reduced risk for myeloma [9], gastric [8,13,14,15], colorectal [6,12], endometrial [7], lung [10], and prostate [11] cancer. Results of one meta-analysis of *Allium* vegetable intake and prostate cancer risk reported significant risk reductions in studies using face-to-face interviews with nutrition experts (odds ratio (OR) = 0.70, 95% confidence interval (CI): 0.59–0.84) compared with those using self-administered food frequency surveys (OR = 0.89, 95% CI: 0.78–1.02) [11]. Likewise, a case-control study employing face-to-face interviews indicated risk ratios of 0.92 (95% CI: 0.79–1.08) and 0.56 (95% CI: 0.44–0.72) for lung cancer corresponding to raw garlic consumption <2 times/week and ≥2 times/week, respectively, compared to individuals who never consumed raw garlic [10]. Consuming 20 g of *Allium* vegetables per day was associated with an OR of 0.91 (95% CI: 0.88–0.94) for gastric cancer [8]. Comparing highest and lowest garlic intakes, two recent meta-analyses reported a risk ratio of 0.49 (95% CI: 0.38–0.62) for gastric cancer [14] and 0.85 (95% CI: 0.72–1.00) for colorectal cancer [12]. A case-control study in France examining development of breast cancer reported an OR of 0.52 (95% CI: 0.34–0.78) among women consuming 7–10 weekly servings of garlic and onions compared to women consuming fewer than six weekly servings [17].

Conversely, there are instances of null findings in some stand-alone cohort studies [18,19] and in meta-analyses evaluations of cohort studies [11,12]. Two cohort studies reporting a null association between garlic intake and colorectal cancer analyzed the Nurses’ Health Study, Health Professionals Follow-Up Study, and Cancer Prevention Study II Nutrition Cohort [18,19]. It is worth noting that these studies used self-administered food frequency questionnaires and considered a serving of garlic as one clove or four shakes of garlic powder or garlic salt [18,19]. It is possible that use of garlic powder and garlic salt within these studies accounted for intake without providing the same protective effect as fresh garlic cloves [18,20]. In one meta-analysis, high and low intakes were considered on such diverse measures as yearly, weekly, and daily consumption as well as subject-assessed ratings of “high” or “low” [14]. Such difference in intake quantification may partially explain the mixed results of different epidemiological studies. Cultural and ethnic differences must also be considered. Greater risk reduction associated with garlic intake was observed in Asian and South American populations than in European populations in some studies [11,14] while similar results across continents were observed in another investigation of total *Allium* vegetable intake [8].

The potential protective effect of garlic supplementation has also been studied. Similar to *Allium* vegetable intake, data regarding garlic supplementation use have shown an inverse association with cancer risk (hazard ratio = 0.55, 95% CI: 0.34–0.87) when comparing high use (≥4 times per week) with non-users [21] as well as null results [18,19,22]. Little description is given about the type of garlic supplement used (powder, aged garlic extract, etc.), which may explain the mixed findings.

## 3. Synthesis, Metabolism, and Pharmacokinetics

**Figure 1:**
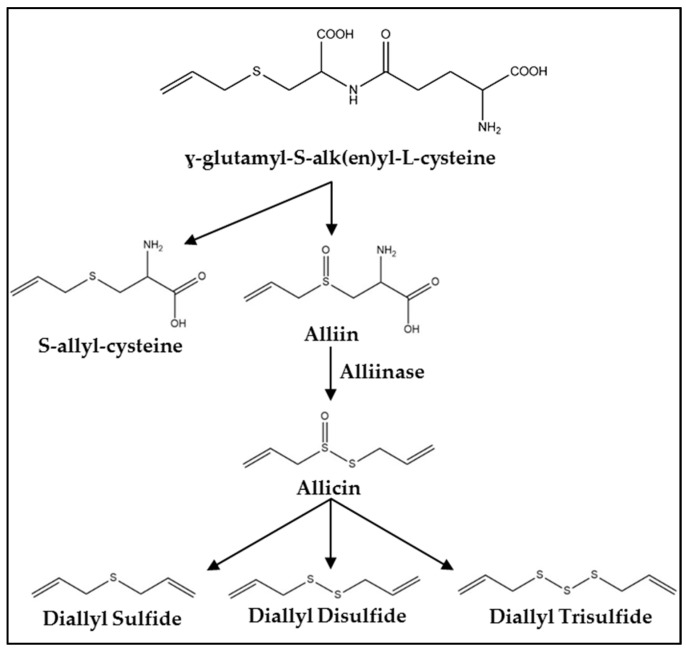
Synthesis pathway of organosulfur compounds (OSCs) in *Allium* vegetables.

Fresh garlic contains a mixture of water, fiber, carbohydrates, protein, and fat as well as more than 20 vitamins and minerals and at least 33 sulfur containing compounds [23,24]. One of the primary precursors of OSCs in garlic is γ-glutamyl-*S*-alk(en)yl-l-cysteine [20] (Figure 1). Within the garlic clove, this compound is hydrolyzed and oxidized into *S*-alk(en)yl-l-cysteine sulfoxide (alliin) which accumulates naturally during storage at cool temperatures [3,20]. Processing garlic by cutting or chewing ruptures vacuoles containing alliinase. This enzyme converts alliin to allicin and other thiosulfinates responsible for the odiferous nature of garlic [3,20]. The allicin yield is about 2.5 mg/g of garlic which correlates to 5–20 mg per clove [23]. Allicin quickly decomposes into products including diallyl sulfide, diallyl disulfide, and DATS with yields of 30–100, 530–610, and 900–1100 μg/g, respectively [20,23].

Pharmacokinetic studies of DATS are limited. In a pharmacokinetic study in rats, following a 10 mg dose of DATS into the jugular vein of rats, DATS plasma concentration peaked at 5.5 μg/mL (31 μM) within 1 min followed by a steady return to baseline levels within 24 h [25]. Using a microemulsion intravenous injection technique in rats to deliver 30 mg/kg DATS, plasma concentration peaked at 7.06 μg/mL (40 μM) within 3 h, indicating a slower clearance rate [26]. Following ingestion of 730 μmol of DATS in human subjects, breath acetone increased reaching a maximum at around 20 h [27]. Allyl methyl sulfide, a known component of breath and putative breakdown product of OSCs, was also increased reaching maximum concentration around the 5 h time point [27]. Further pharmacokinetic studies of DATS are vital for its investigation as a chemopreventive agent. In order to achieve accurate results, compound stability must also be considered. Stability of DATS in rat blood was shown to be influenced by purification technique, storage temperature, duration, and freeze–thaw cycles [25].

## 4. Mechanisms of Action

**Figure 2:**
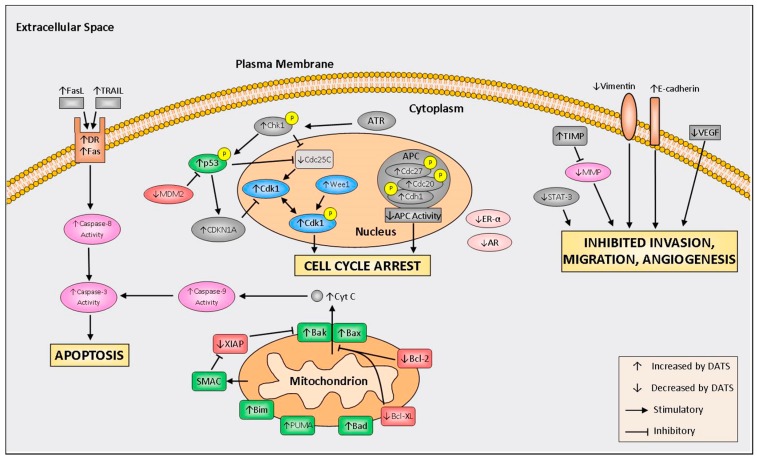
Summary of molecular mechanisms regulated by diallyl trisulfide (DATS). FasL, Fas ligand; TRAIL, TNF-related apoptosis-inducing ligand; DR, death receptor; ATR, ataxia telangiectasia and Rad3-related protein; MDM2, mouse double minute 2 homolog; Chk1, checkpoint kinase 1; CDKN1A, cyclin dependent kinase inhibitor 1A; Cdk1, cyclin-dependent kinase 1; Cdc25c, cell division cycle 25C; APC, anaphase promoting complex; XIAP, X-linked inhibitor of apoptosis protein; SMAC, second mitochondria-derived activator of caspases; Bim, Bcl-2-like protein 11; PUMA, p53 upregulated modulator of apoptosis; Bad, Bcl-2-associated death promoter; Bcl-XL, B-cell lymphoma-extra large; Bcl-2, B-cell lymphoma 2; Bax, bcl-2-like protein 4; Bak, Bcl-2 homologous antagonist; ER-α, estrogen receptor alpha; AR, androgen receptor; TIMP, tissue inhibitor of metalloproteinase; MMP, matrix metalloproteinase; STAT-3, signal transducer and activator of transcription 3; and VEGF, vascular endothelial growth factor.

Much of the research into the DATS mechanisms of action refers to induction of cell cycle arrest, increased programmed cell death, inhibition of invasion, migration, and reduced angiogenesis. A schematic of molecular mechanisms of DATS is depicted in Figure 2. A summary of in vivo studies is shown in Table 1.

### 4.1. Cell Cycle Arrest

Deregulation of cell cycle checkpoint mechanisms is an initiating event in cancer development allowing for uncontrolled cell cycle progression and rapid tumor growth. Several studies have shown that DATS induces G2/M phase cell cycle arrest [28,29,30,31,32,33,34,35,36,37,38,39,40,41,42,43,44,45,46]. DATS has been shown to induce cell cycle arrest by enhancing generation of reactive oxygen species (ROS) [33,34,43,45,47]. One pathway of ROS generation begins with degradation of the iron storage protein ferritin, which has been shown following DATS treatment, leading to an increase in labile iron pool (LIP) size [43,47,48]. Then, through the Fenton/Haber-Weiss reaction, free ferric iron reacts with superoxide and hydrogen peroxide to form hydroxyl radicals and hydroxide ions [34,49]. These and other ROS are involved in biological processes such as DNA damage and cell death signal transduction [49]. ROS induced DNA damage may explain the rapid phosphorylation and activation of the DNA damage sensing protein checkpoint kinase 1 (Chk1) [39]. In Chk1 expressing prostate cancer cells, DATS induced accumulation of CyclinB1, securin, and serine 10 phosphorylated Histone H3 (p-H3) corresponding to mitotic arrest [39]. Cells expressing wild type ataxia telangiectasia and Rad3 related (ATR), another DNA damage sensing protein, showed similar accumulation [35]. Prostate and colon cancer cells displayed less accumulation of these targets following Chk1 knockdown and expression of inactive ATR, respectively [35,39]. Together, these data suggest that DATS-induced accumulation of cells in G2/M may be partially dependent on DNA damage checkpoint proteins Chk1 and ATR [35,39].

DATS treatment has been shown to regulate downstream targets of another DNA damage sensing protein, p53, including cell division cycle 25C protein (Cdc25C), cyclin-dependent kinase 1 (Cdk1), and Wee1 [31,33,34,38,39,40,42]. These proteins are well characterized cell cycle regulators further supporting the idea that DATS induces cell cycle arrest through induction of DNA damage. Cdc25C, a regulatory phosphatase that promotes passage into mitosis, has been well characterized [34,40]. The reduced Cdc25C protein levels triggered by DATS were reversed with antioxidant pre-treatment suggesting oxidative stress may account for decreased expression [34,40]. This finding is consistent with data indicating that oxidation of cysteine residues at amino acids 330 and 377 negatively impacted Cdc25C stability [65]. In contrast to these findings, overexpression of wild type Cdc25C as well as expression of protein mutated at key cysteine residues failed to rescue prostate cancer cells from DATS-induced G2/M arrest suggesting a dispensable role of Cdc25C in this model [40].

DATS-mediated arrest during passage into mitosis has been examined by several investigators [28,31,33,35,38,39,40,42,46,66,67]. During the G2/M transition, Cdc25C mediated dephosphorylation of Cdk1 and subsequent Cdk1/CyclinB1 complex formation promote entrance into mitosis as a result of enhanced kinase activity. Although DATS prompted an increase in CyclinB1 protein, treatment also reduced Cdc25C expression and increased levels of tyrosine 15 phosphorylated Cdk1 leading to inactivation of the Cdk1/CyclinB1 complex [28,31,33,35,38,39,42,46,66,67]. Without adequate levels of Cdk1 and CyclinB1 co-localized in the nucleus, cells are unable to move past the G2/M checkpoint. Following the start of DATS treatment, CyclinB1 appeared in the nucleus within one hour while Cdk1 was not observed in the nucleus until between hours 2 and 4 [40]. These findings may clarify the transient nature of G2/M arrest as cells eventually regained Cdk1 kinase activity after 8 hours of DATS treatment [40]. A small percentage of treated cells have shown the ability to escape G2/M arrest during 24 h of continuous DATS treatment [40]. Another group detailed gradual return to normal cell cycle following cessation of a 12 h DATS treatment [36].

Mitotic exit is dependent on an active anaphase promoting complex (APC), containing Cdc27, Cdc20, and Cdh1 subunits [39]. DATS promoted inactivation of Cdc27 and Cdh1 by maintenance of phosphorylation [35,39]. Similarly, phosphorylated Cdh1 and Cdc20 were more prevalent in ATR wild type cells than in cells expressing inactive ATR [35]. The APC substrate securin, which must be degraded for sister chromatids to separate, was increased following DATS incubation [35,39]. This is thought to be a result of decreased APC activity rather than up-regulation of securin [35,39]. After DATS treatment, staining of α-tubulin showed fluorescence exclusively surrounding the nucleus rather than throughout the whole cell as observed in control cells [33,39]. These data agree with the finding that DATS disrupted the microtubule network in colon cancer cells [28,36]. Taken together, these results suggest DATS may also induce G2/M arrest by reducing the ability of chromosomes to separate during the mitotic phase of the cell cycle.

While multiple reports indicate no change in the fraction of S phase arrested cells [66,67], evidence exists supporting both increased [64,66,68] and decreased [33,38,39,67] G0/G1 fractions following DATS treatment.

Increased cell cycle arrest following DATS treatment was recently connected to reduced histone deacetylase activity (HDAC) and enhanced histone H3 and H4 acetylation in vivo [63]. Persistent acetylation surrounding negative regulators of the cell cycle may diminish checkpoint passage thereby inhibiting cell cycle progression [63]. Enhanced p53 expression, MDM-2 degradation, reduced Cdc25C, increased cyclin dependent kinase inhibitor 1A, (CDKN1A, p21^Cip1/Waf1^), and increased p-Cdk1 induced G2/M arrest in glioblastoma tumors after 1 week of DATS treatment [63]. Increased CyclinB1 and securin by DATS further support a large body of evidence suggesting DATS promotes cell cycle arrest in vivo [46,51].

### 4.2. Induction of Apoptosis

Enhancing apoptosis is a promising anti-cancer strategy aimed at reducing tumor progression. A growing body of evidence suggests that DATS acts by prompting cancer cells to obey cell death signals including DNA damage, oxidative stress, and cellular damage.

In addition to its role in cell cycle arrest, generation of ROS is known to induce cell death [37,42,57,69,70,71,72]. As mentioned previously, ferritin degradation and subsequent increased LIP size is one mechanism by which ROS are generated following DATS treatment. Augmented expression of p66Shc and Itch E3 ligase induced by DATS are part of the mechanism by which ferritin is degraded [43,47,48,73]. These proteins are further implicated in cell death signal propagation as their reduced wild type expression resulted in resistance to the effects of DATS [43,48]. The role of LIP size in DATS-mediated cell death recently came into question by the finding that iron chelation afforded no protection from DATS-induced cytotoxicity [48]. Further research is required to characterize the role of iron in cancer cells as high iron status may have a substantial impact on promoting neoplastic progression [74].

Results across many cancer cell types have reported activation of the intrinsic apoptotic pathway following DATS treatment. Increased activation of the apoptosis regulating protein c-Jun N-terminal kinase (JNK) has been shown across many studies after DATS incubation [37,57,69,75,76,77,78]. One cause of JNK activation may be related to generation of another potent ROS, hydrogen peroxide. Under non-stress conditions the JNK activator apoptosis signal-regulating kinase 1 (ASK1) is held inactive by the ubiquitous redox sensing proteins thioredoxin (TRX) and glutaredoxin (GRX). Certain stressors promote oxidation of the intermolecular disulfide bond between TRX and GRX causing dissociation and subsequent activation of ASK1. Enhanced ASK1 dissociation in addition to JNK phosphorylation following DATS treatment were observed in breast cancer cells [77]. Furthermore, ASK1 activation was inhibited following catalase transfection implicating DATS-induced production of hydrogen peroxide as the putative source of TRX:GRX bond oxidation [77].

Increased expression of p53 has been shown to have pro-apoptotic action in pancreatic [67], lung [37], breast [66], and skin [42,45] cancer cells. DATS treatment increased nuclear translocation of p53 in breast and pancreatic cancer cells and decreased expression of MDM2, a negative regulator of p53 [66,67]. Downstream targets of p53 include members of the B-cell lymphoma 2 (Bcl-2) family of proteins involved in regulating intrinsic apoptosis [79]. Thus, DATS treatment is known to increase apoptosis by regulating expression of pro-apoptotic Bcl-2-like protein 4 (Bax) [38,41,44,45,46,50,61,66,67,71,72,78,80], Bcl-2 homologous antagonist (Bak) [38,50,70,72], BH3 interacting domain death agonist (Bid) [38], Bcl-2-associated death promoter (Bad) [57], p53 upregulated modulator of apoptosis (PUMA) [50,80], and phorbol-12-myristate-13-acetate-induced protein 1 (NOXA) [50] as well as anti-apoptotic Bcl-2 [37,38,41,44,45,50,57,61,66,67,70,71,76,78,80,81,82] and B-cell lymphoma-extra large (Bcl-XL) [38,45,50,70,78,82]. Moreover, the pro-apoptotic protein Bcl-2-like protein 11 (Bim), which is a downstream target of JNK, displayed increased phosphorylation following DATS incubation thus providing one connection between DATS treatment, ROS generation, and induction of apoptosis [50,77]. It is noteworthy that, although Bcl-2 overexpression did not provide protection from DATS-induced apoptosis in one prostate cancer cell line, Bax and Bak knockdown conferred partial resistance [38,70]. These data suggest DATS-mediated apoptosis may be attributed to enhanced pro-apoptotic action of Bax and Bak rather than to reduced anti-apoptotic activity of Bcl-2 and Bcl-XL. Results show that enhanced apoptotic action may be brought about by translocation of pro-apoptotic proteins to the mitochondrial membrane due to disruption of the interaction with Bcl-2 [45,76] and decreased interaction with 14-3-3 proteins [83].

Localization of pro-apoptotic proteins to the mitochondrial membrane leads to mitochondrial membrane depolarization [37,42,45,50,57,69,70,71,78], calcium release from the endoplasmic reticulum [42,45,69,84], and mitochondrial release of various apoptosis regulating proteins. Studies investigating DATS-induced apoptosis consistently support the decrease of X-linked inhibitor of apoptosis protein (XIAP) expression [46,50,52,78] following DATS treatment, but cell specific discrepancies exist regarding regulation of cIAP [50,52,78] and survivin [37,50,52]. Following release from the mitochondria, cytochrome C is known to complex with Apaf-1 to initiate cleavage of pro-caspase-9 thus activating caspase-dependent apoptosis through executioner caspase-3. Reports of enhanced cytochrome C release [30,45,50,61,69,70,71,78,82], increased Apaf-1 protein [45], enhanced caspase-9 expression and activity [42,45,50,61,78,82], and greater cleaved poly (ADP-ribose) polymerase (PARP) [38,41,42,45,57,78,82,83] further support the extensive pro-apoptotic capabilities of DATS. Many pro-apoptotic effects have been shown to be partially abolished following superoxide dismutase overexpression [72] or pre-treatment with an antioxidant such as N-acetylcysteine [37,45,57,70] suggesting they are ROS dependent.

In addition to the intrinsic apoptotic mechanism, DATS is known to affect the extrinsic apoptosis pathway. Extrinsic apoptosis is initiated through binding of an extracellular death ligand such as TNF-related apoptosis-inducing ligand (TRAIL) or Fas to a death receptor (DR) on the cell membrane. The subsequent signaling cascade results in caspase-8 activation which also activates caspase-3. LNCaP prostate cancer cells have been reported to be resistant to TRAIL-induced apoptosis while PC-3 cells are TRAIL sensitive [50]. DATS treatment was reported to sensitize LNCaP cells to TRAIL-induced apoptosis and synergize with TRAIL to enhance apoptosis in PC-3 cells [50]. Greater expression of extrinsic pathway constituents such as DRs as well as their ligands has been observed in other cancer types following DATS treatment [37,66,67,78]. Additionally, downstream events including increased activity of caspase-8 [50,61,78,85] and caspase-3 [28,30,39,42,45,46,50,61,64,69,70,71,78,81,82] have been reported. Components of the endoplasmic reticulum stress-mediated apoptosis pathway including BiP, CHOP, and caspase-4 were shown to be increased in one study following DATS treatment [45]. Addition of a caspase inhibitor did not completely reverse the effects of DATS in a basal cell carcinoma model implying apoptotic action also occurs through caspase independent pathways [45].

Results from in vitro studies have shown DATS to be more potent at reducing cell viability and proliferation than other OSCs including diallyl disulfide and diallyl sulfide [38,42,57,58,59,84,86]. Investigation of nine trisulfides found those with three carbon chains to provide the most inhibition of cell viability compared to compounds with longer chains [87]. It is important to note that DATS has been reported to have lower toxicity to non-transformed cells as compared to cancer cells at similar concentrations [33,38,47,70,72,84]. Though some levels of ROS were generated in the normal breast epithelial cell line MCF-10A following DATS incubation, Bax and Bak expression as well as apoptotic figures remained unchanged [72]. While these data provide an explanation for how MCF-10A cells avoid apoptosis, further mechanistic studies are needed to elucidate how these cells avoid altering apoptosis-related gene expression in spite of ROS production. Such research may provide insight into how transformed cells evade apoptosis during cancer progression and promote understanding of the mechanisms by which normal cells are protected from the effects of DATS.

Several in vivo studies in various cancer models reported decreased tumor volumes as well as lower tumor incidence and multiplicity following DATS treatment [46,50,52,55,56,57,58,61,62,63,88]. The DNA binding ability of transcription factor AP-1 and activity of cell survival pathway constituent Akt were shown to be reduced following DATS treatment [62]. Additional regulation of c-Myc, mechanistic target of rapamycin (mTOR), tumor necrosis factor α (TNF-α), interleukin 6 (IL-6), IκB kinase (IKK), IκB-α, and NFκB expression observed in multiple models correspond to additional inhibition of survival pathways as a result of DATS treatment [46,50,51,62]. Topical application of DATS (25 μmol) prior to application of a carcinogen was shown to attenuate expression of prostaglandin-endoperoxide synthase 2, also as cyclooxygenase-2 (COX-2) [62]. These results support the notion that DATS may act as an anti-cancer agent in vivo by inhibiting cancer progression.

Xenograft glioblastoma tumors displayed enhanced apoptosis through reduced Bcl-2 expression, increased Bax protein levels, and caspase-3 activation [63]. DATS treatment of orthotopically implanted prostate cancer cells in nude mice revealed an increased pro-apoptotic:anti-apoptotic signal ratio by modulation of Bax, Bak, Bcl-2, and Bcl-XL along with increased DR protein levels [50]. In a TRAMP model of prostate cancer, decreased XIAP protein along with increased survivin have been documented following DATS administration [52]. 

While hematopoietic cancers do not display solid tumors, reduced cancer progression was evident following DATS treatment of nude mice intraperitoneally injected with murine leukemia cells [64]. Enhanced phagocytic activity and natural killer (NK) cell cytotoxicity were observed in DATS treated mice following 10 mg/kg treatment for 14 days [64]. In cancer bearing mice, B-cell proliferation was reduced with DATS treatment [64]. Non-leukemic mice showed no change in phagocytosis or B-cell proliferation representing another instance of the relative innocuous nature of DATS toward un-transformed cell types [64].

### 4.3. Inhibition of Invasion, Migration, and Angiogenesis

A cancer diagnosis in late stages, after metastasis has occurred, represents up to a 13.5 fold decrease in survival compared to diagnosis during early, localized stages [1]. These statistics make apparent the need for anti-cancer strategies aimed at reducing invasion and migration. Recent studies have documented inhibition of migration and invasion-related proteins following DATS treatment. Expression and activity of matrix metalloproteinase (MMP)-2, -7, and -9 were inhibited indicating decreased ability to degrade basement membranes [55,59,68,86,89]. Known inhibitors of MMPs, TIMP-1 and -2, were increased upon DATS treatment leading to enhanced tight junction formation between bladder cancer cells [89]. Claudin expression was shown to decrease following DATS treatment [89]. Expression of different claudins and their relation to an invasive phenotype are highly contextual as lines of evidence exist claiming claudins both promote [90] and inhibit [91,92] migration, invasion, and metastasis. These results require further research to elucidate the cell-specific relationship between claudin expression and overall invasiveness. While enhanced JNK1/2 expression was shown to be responsible for apoptosis induction [37,57,76,77], others have shown JNK2 to enhance cell migration [93]. Further research is required to better characterize this apparent dichotomy. Cell surface proteins vimentin and E-cadherin, which are also involved in the epithelial-mesenchymal transition, were regulated following DATS treatment to favor increased adhesion [72]. Regulation of the Janus kinase (JAK)/signal transducer and activator of transcription (STAT) pathway may also contribute to migration and invasion of cancer cells as ectopic expression of STAT3 in LNCaP cells resulted in enhanced migration [53]. DATS treatment reduced migration by blocking STAT3 phosphorylation [53].

DATS treatment has been shown to reduce expression and secretion of vascular endothelial growth factor (VEGF) in several cancer cell types [55,68]. Human umbilical vein endothelial cells treated with DATS demonstrated decreased VEGF secretion, decreased capillary-like tube formation, and reduced VEGF receptor expression [55,94]. Incubation of endothelial cells with conditioned media from DATS-treated osteosarcoma cells resulted in decreased capillary-like tube formation compared to incubation with media from untreated cells [68].

In vivo studies reported effects of DATS-mediated invasion, migration, and angiogenesis. A study in chick embryos documented reduced angiogenesis following DATS treatment [55]. This supports prior findings that four weeks of daily 40 mg/kg DATS decreased microvessel density along with VEGF and IL-6 expression [50]. In a glioblastoma xenograft model, VEGF expression was reduced following seven days of DATS treatment [63]. Decreased hemoglobin concentration in tumor sections was observed in colon cancer xenograft studies with 50 mg/kg DATS suggesting anti-angiogenic action [55,56]. However, a 2 mg DATS treatment of TRAMP mice did not decrease number of vessels, diameter of vessels, or expression of angiogenesis marker CD31 [51]. These results indicate that, while DATS treatment may directly influence angiogenesis in some studies, cancer type and dosage must be considered.

Decreased expression of MMP-2, -7, and -9, and p-STAT3 was observed in mouse models supporting the role of DATS as an inhibitor of invasion and migration [50,53]. Decreased incidence of poorly differentiated prostate carcinoma in TRAMP mice was observed in dorsolateral prostates following treatment with 1 and 2 mg DATS [51]. These data suggest DATS may be able to inhibit the transformation from a well differentiated to poorly differentiated phenotype and reduce lung and lymph node metastases [51]. In a zebrafish model, DATS treatment decreased the number of metastatic foci as well as maximal metastatic distance of triple negative breast cancer cells [59].

### 4.4. Modulation of Hormone Regulated Cancers

Hormone signaling by estrogen and androgens is known to play an important role in progression of breast and prostate cancers, respectively. In breast cancer, estrogen sensitivity and HER-2 expression are important factors in patient prognosis. Studies involving breast cancer cell lines differing in estrogen sensitivity and HER-2 status showed diminished cell viability upon DATS treatment [72,95]. The observed reduction in cell viability was unaltered following ER-α overexpression in the triple negative breast cancer cell line MDA-MB-231 [95]. These data suggest DATS as an attractive anti-cancer agent because of its broad ability to decrease cell viability in breast cancer cells regardless of ER-α and HER-2 status. DATS treatment also reduced estrogen receptor mRNA, protein levels, and reporter activity in ER-α positive breast cancer cell lines MCF-7 and T47D [95].

In prostate cancer, the effect of androgen dependence has been investigated by comparing the effect of DATS in androgen independent PC-3 and androgen dependent LNCaP cell lines. Both underwent significant apoptosis following DATS treatment [70,76]. In addition, DATS reduced androgen receptor mRNA level, protein level, transcriptional activity, and PSA secretion in prostate cancer cells [54]. Furthermore, DATS reversed androgen receptor nuclear translocation and cell proliferation induced by the androgen analog R1881 [54].

### 4.5. Other Mechanisms

Reduction of chemically induced carcinogenesis was among the first anti-cancer strategies investigated using DATS and this has been reviewed by others [16,96]. Briefly, studies investigating the activity of chemical metabolizing enzymes in mice following DATS incubation showed suppression of phase I enzymes responsible for activation of chemical carcinogens and induction of phase II enzymes responsible for detoxification [97]. DATS was shown to induce phase II enzymes NADPH:quinone oxireductase and heme oxygenase [60,98]. Induction of phase II enzymes is a desirable goal especially in preventing cancer initiating events as cells are able to better inactivate and remove mutation causing carcinogens [99]. The stress-sensing protein nuclear factor erythroid-2-related factor 2 (Nrf2) and the antioxidant response axis may be partially responsible for this observed effect. In one study, Nrf2 expression was positively correlated with expression of multiple phase II genes [75] while another study observed reversal of phase II gene induction in DATS treated cells transfected with Nrf2 siRNA [60]. Under non-stress conditions, Nrf2 is degraded in the cytoplasm through interaction with Keap1. Introduction of stressors modifies cysteine residues on Keap1 resulting in Nrf2 accumulation, translocation to the nucleus, and induction of downstream targets. Increased Nrf2 protein levels and augmented antioxidant response element activity have been observed in liver cancer cells following DATS treatment [75]. In fact, DATS may directly be involved in Nrf2 signaling as the mass of a mono-allyl sulfide moiety was recently observed bound to the protein fragment containing cysteine 288 of Keap1 following DATS treatment of gastric cancer cells [60]. This amino acid was shown to be necessary for regulating Nrf2 activity as a mutation resulted in failed induction of target genes [60].

Altered cellular signaling of key pathways involved in growth, differentiation, and development exemplify the breadth of mechanisms affected by DATS. Inhibition of mTOR, NF-κB, and MAPK signaling cascades provide further evidence that DATS limits the survival capacity of cancer cells [37,41,43,46,47,57,59,60,66,73,76,78]. HDAC inhibition as well as increased acetylation of the promoter for IκB-α regulator MT2A in gastric cancer cells provides one mechanism by which DATS may regulate NF-κB [46]. The Notch developmental and cancer stem cell-related pathway has been shown to be inhibited by DATS in osteosarcoma cells [68]. Decreased levels of Notch-1, Hes, and Hey were reported upon DATS incubation along with reduced downstream expression of CyclinD1 [68]. The same study observed increased expression of miR-143 and miR-145 and decreased expression of miR-21 following DATS treatment [68]. In another cancer stem cell-related study, DATS reduced the CD44^high^/CD24^low^/ESA^+^ population as well as aldehyde dehydrogenase (ALDH) activity in breast cancer cells [58]. More recently, DATS was shown to inhibit Notch ligands and alpha secretases in breast cancer cells [100].

Production of hydrogen sulfide (H_2_S) has been proposed to mediate the beneficial effects of dietary OSCs such as DATS. DATS acts as a H_2_S donor upon reaction with glutathione [101]. H_2_S has cardioprotective effects and beneficial effects against liver fibrosis [102,103]. In addition, H_2_S donors have been proposed to act as anti-cancer drugs [104,105,106]. While endogenously produced H_2_S may enhance tumor growth, higher concentrations of exogenous H_2_S (from a H_2_S donor) could lead to suppression of tumor growth by inducing intracellular acidification, cell cycle arrest, and apoptosis [104,105,106]. H_2_S contribution to DATS-induced suppression of tumor growth warrants further investigation.

Studies of combination treatments of DATS and chemotherapeutic drugs have investigated DATS as an adjuvant therapy [46,107]. Combination treatments of DATS and docetaxel [46] or cisplatin [107] reported synergistic inhibition of gastric cancer cell growth. DATS sensitized gastric cancer cells to docetaxel through the metallothionein 2A/NF-κB pathway, and enhanced G2/M phase cell cycle arrest and apoptosis [46]. In xenograft animal models with gastric cancer cells, the combination of DATS and either docetaxel [46] or cisplatin [107] resulted in greater tumor growth inhibition when compared to the groups that received either drug alone.

## 5. Clinical Studies

Multiple human trials have been undertaken to investigate the anti-cancer capabilities of garlic-derived compounds [108,109,110,111,112,113], but only one clinical study has specifically examined the anti-cancer role of DATS [114]. A combination of 200 mg synthetic DATS every day along with 100 μg selenium every other day was administered for one month per year for three years in a Chinese population at moderate to high risk for developing gastric cancer based on presence of stomach disorders, family history of cancer, or lifestyle factors such as smoking or alcohol consumption [114]. In the first five years of follow-up, the relative risk in the whole intervention group was 0.67 (95% CI: 0.43–1.03) for malignant tumors and 0.48 (95% CI: 0.21–1.06) for gastric cancer [114]. The relative risk associated with the DATS intervention reached significance for malignant tumors (RR = 0.51 (95% CI: 0.30–0.85)) and gastric cancer (RR = 0.36 (95% CI: 0.14–0.92)) in men only. The study also reported that this dose of synthetic DATS was well tolerated. Future clinical trials investigating the effects of DATS are needed. Dose, duration, cancer type, and cancer stage must be carefully considered to determine the anti-cancer and chemopreventive efficacy of DATS.

## 6. Conclusions and Future Perspective

Epidemiological studies provide background support for the beneficial health properties of garlic, known by humans for centuries, by correlating *Allium* vegetable intake with reduced cancer risk. Accumulating evidence has shown DATS to regulate several cancer-related pathways including cell cycle arrest, apoptosis, chemical detoxification, invasion, migration, and angiogenesis. Many similar outcomes from cell-based and preclinical models document the molecular mechanisms involved in the anti-cancer effects of DATS and provide rationale for future investigation. Pre-clinical in vivo studies are needed to document the effect of DATS at different stages of carcinogenesis. Future studies of DATS pharmacokinetics and metabolism are necessary to better support the design and execution of clinical studies.

## Figures and Tables

**Table 1:** Summary of in vivo mechanisms of action of DATS.

Cancer Type	Model (Organism/Cell Line)	Dose	Effect/Mechanism	Reference
Prostate	BALB/c orthotopic/PC-3	40 mg/kg; 5× per week; 4 weeks	Inhibited tumor growth, migration, invasion, angiogenesis, induced apoptosis; ↑ DR4/5; ↓ Bcl-2; ↓ Bcl-XL; ↑ Bax; ↑ Bak; ↓ IKK activity; ↓ p-Akt; ↓ MMP-2/7/9; ↓ VEGF; ↓ IL-6;	[50]
	TRAMP	2 mg; 3× per week; 13 weeks	Inhibited cancer progression, ↓ poorly differentiated carcinoma; ↓ pulmonary and pelvic lymph node metastases; ↓ neuroendocrine differentiation; induced apoptosis; inhibited migration and invasion; ↑ cyclinB1; ↑ securin; ↓ XIAP; ↓ STAT-3; ↓ p-STAT3; ↓ androgen receptor	[51,52,53,54]
Colon	BALB/c xenograft/HT-29	50 mg/kg; daily; 4 weeks	Inhibited tumor growth; reduced angiogenesis	[55]
	BALB/c allograft/CT26	50 mg/kg; every 4 days; 32 days	Inhibited tumor growth; reduced angiogenesis	[56]
Breast	BALB/c xenograft/MCF-7	5 μmol/kg; 2× per week; 1 month	Inhibited tumor growth; ↓ tumor volume	[57]
	SCID xenograft/SUM159	2 mg; 3× per week; 55 days	Reduced stemness; ↓ tumor incidence; ↓ ALDH activity	[58]
	Zebrafish/MDA-MB-231	10–20 μM for 24 h	Inhibited migration and invasion; ↓ metastatic foci; ↓ maximal metastatic distance	[59]
Gastric	C57BL/6	0.5 and 2 mg/kg; every other day; 2 weeks	↑ Nrf2; ↑ NQO-1; ↑ HO-1	[60]
	BALB/c xenograft/BGC823	20 mg/kg; every 4 days; 20 or 24 days	Inhibited tumor growth; induced G2/M arrest; induced apoptosis; ↑ IκB-α; ↑ CyclinB1; ↓ CyclinD1;	[46]
Lung	BALB/c xenograft/A549	6 μM (100 μl); every other day; 30 days	Inhibited cancer progression; ↓ tumor incidence; ↓ tumor volume	[61]
Skin	ICR DMBA initiated papillomas	25 μmol topically prior to TPA application	Reduced tumor incidence and multiplicity; ↓ COX-2; ↓ AP-1 DNA binding; ↓ JNK activity; ↓ Akt activity;	[62]
Glioblastoma	NOD/Prkdcscid/J xenograft/U87MG	10 μg/kg–10 mg/kg; daily; 1 week	Inhibited tumor growth, induced G2/M arrest, apoptosis; ↓ HDAC activity; ↑ histone H3/4 acetylation; ↓ Cdk1; ↓ Cdc25C; ↑ CDKN1A (p21^Cip1/Waf1^); ↑ p53; ↓ p-Akt; ↓ c-Myc; ↓ mTOR; ↓VEGF; ↑ Bax; ↓ Bcl-2;	[63]
Leukemia	BALB/c xenograft/WEHI-3	10 mg/kg; daily; 2 weeks	Inhibited cancer progression; ↑ macrophage activity; ↑ NK cell activity; ↓ B-cell proliferation	[64]

DR4/5, death receptor 4/5; Bcl-2, B-cell lymphoma 2; Bcl-XL, B-cell lymphoma-extra large; Bax, Bcl-2-like protein 4; Bak, Bcl-2 homologous antagonist; IKK, IκB kinase; p-Akt, phospho-protein kinase B; MMP-2/7/9, matrix metalloproteinase-2/7/9; VEGF, vascular endothelial growth factor; IL-6, interleukin 6; XIAP, X-linked inhibitor of apoptosis protein; STAT-3, signal transducer and activator of transcription 3; ALDH, aldehyde dehydrogenase; Nrf2, nuclear factor erythroid-2-related factor 2; NQO-1, NAD(P)H: quinone oxidoreductase-1; HO-1, heme oxygenase-1; IκB-α, nuclear factor of kappa light polypeptide gene enhancer in B-cells inhibitor, alpha; COX-2, cyclooxygenase-2; AP-1, activator protein 1; JNK, c-Jun N-terminal kinase; HDAC, histone deacetylase; Cdk1, cyclin-dependent kinase 1; Cdc25C, cell division cycle 25C; CDKN1A, cyclin dependent kinase inhibitor 1A; mTOR, mechanistic target of rapamycin; VEGF, vascular endothelial growth factor; and NK, natural killer.

## References

[1] Bray F., Jemal A., Grey N., Ferlay J., Forman D. (2012). Global cancer transitions according to the human development index (2008–2030): A population-based study. Lancet Oncol..

[2] Siegel R.L., Miller K.D., Jemal A. (2017). Cancer statistics, 2017. CA Cancer J. Clin..

[3] Block E. (1985). The chemistry of garlic and onions. Sci. Am..

[4] Petrovska B.B., Cekovska S. (2010). Extracts from the history and medical properties of garlic. Pharmacogn. Rev..

[5] Mikaili P., Maadirad S., Moloudizargari M., Aghajanshakeri S., Sarahroodi S. (2013). Therapeutic uses and pharmacological properties of garlic, shallot, and their biologically active compounds. Iran. J. Basic Med. Sci..

[6] Millen A.E., Subar A.F., Graubard B.I., Peters U., Hayes R.B., Weissfeld J.L., Yokochi L.A., Ziegler R.G., PLCO Cancer Screening Trial Project Team (2007). Fruit and vegetable intake and prevalence of colorectal adenoma in a cancer screening trial. Am. J. Clin. Nutr..

[7] Galeone C., Pelucchi C., Dal Maso L., Negri E., Montella M., Zucchetto A., Talamini R., La Vecchia C. (2009). *Allium* vegetables intake and endometrial cancer risk. Public Health Nutr..

[8] Zhou Y., Zhuang W., Hu W., Liu G., Wu T.X., Wu X.T. (2011). Consumption of large amounts of *Allium* vegetables reduces risk for gastric cancer in a meta-analysis. Gastroenterology.

[9] Wang Q., Wang Y., Ji Z., Chen X., Pan Y., Gao G., Gu H., Yang Y., Choi B.C., Yan Y. (2012). Risk factors for multiple myeloma: A hospital-based case-control study in Northwest China. Cancer Epidemiol..

[10] Jin Z.Y., Wu M., Han R.Q., Zhang X.F., Wang X.S., Liu A.M., Zhou J.Y., Lu Q.Y., Zhang Z.F., Zhao J.K. (2013). Raw garlic consumption as a protective factor for lung cancer, a population-based case-control study in a Chinese population. Cancer Prev. Res..

[11] Zhou X.F., Ding Z.S., Liu N.B. (2013). *Allium* vegetables and risk of prostate cancer: Evidence from 132,192 subjects. Asian Pac. J. Cancer Prev..

[12] Turati F., Guercio V., Pelucchi C., La Vecchia C., Galeone C. (2014). Colorectal cancer and adenomatous polyps in relation to *Allium* vegetables intake: A meta-analysis of observational studies. Mol. Nutr. Food Res..

[13] Woo H.D., Park S., Oh K., Kim H.J., Shin H.R., Moon H.K., Kim J. (2014). Diet and cancer risk in the Korean population: A meta-analysis. Asian Pac. J. Cancer Prev..

[14] Kodali R.T., Eslick G.D. (2015). Meta-analysis: Does garlic intake reduce risk of gastric cancer?. Nutr. Cancer.

[15] Turati F., Pelucchi C., Guercio V., La Vecchia C., Galeone C. (2015). *Allium* vegetable intake and gastric cancer: A case-control study and meta-analysis. Mol. Nutr. Food Res..

[16] Antony M.L., Singh S.V. (2011). Molecular mechanisms and targets of cancer chemoprevention by garlic-derived bioactive compound diallyl trisulfide. Indian J. Exp. Biol..

[17] Challier B., Perarnau J.M., Viel J.F. (1998). Garlic, onion and cereal fibre as protective factors for breast cancer: A French case-control study. Eur. J. Epidemiol..

[18] McCullough M.L., Jacobs E.J., Shah R., Campbell P.T., Gapstur S.M. (2012). Garlic consumption and colorectal cancer risk in the CPS-II Nutrition Cohort. Cancer Causes Control.

[19] Meng S., Zhang X., Giovannucci E.L., Ma J., Fuchs C.S. (2013). No association between garlic intake and risk of colorectal cancer. Cancer Epidemiol..

[20] Amagase H., Petesch B.L., Matsuura H., Kasuga S., Itakura Y. (2001). Intake of garlic and its bioactive components. J. Nutr..

[21] Walter R.B., Brasky T.M., Milano F., White E. (2011). Vitamin, mineral, and specialty supplements and risk of hematologic malignancies in the prospective VITamins and Lifestyle (VITAL) study. Cancer Epidemiol. Biomark. Prev..

[22] Heine-Bröring R.C., Winkels R.M., Renkema J.M., Kragt L., van Orten-Luiten A.C., Tigchelaar E.F., Chan D.S., Nora T., Kampman E. (2015). Dietary supplement use and colorectal cancer risk: A systematic review and meta-analyses of prospective cohort studies. Int. J. Cancer.

[23] Shukla Y., Kalra N. (2007). Cancer chemoprevention with garlic and its constituents. Cancer Lett..

[24] Li J., Liu W., Zhao K., Zhang Y., Li X., Yang Q., Li Z., Li J. (2009). Diallyl trisulfide reverses drug resistance and lowers the ratio of CD133+ cells in conjunction with methotrexate in a human osteosarcoma drug-resistant cell subline. Mol. Med. Rep..

[25] Sun X., Guo T., He J., Zhao M., Yan M., Cui F., Deng Y. (2006). Determination of the concentration of diallyl trisulfide in rat whole blood using gas chromatography with electron-capture detection and identification of its major metabolite with gas chromatography mass spectrometry. Yakugaku Zasshi.

[26] Li X., Yue Y., Zhou Y., Fan Y., Fan C., Huang Y., Wu F., Liu Y. (2011). An oil-free microemulsion for intravenous delivery of diallyl trisulfide: Formulation and evaluation. Int. J. Pharm..

[27] Lawson L.D., Wang Z.J. (2005). Allicin and allicin-derived garlic compounds increase breath acetone through allyl methyl sulfide: Use in measuring allicin bioavailability. J. Agric. Food Chem..

[28] Hosono T., Fukao T., Ogihara J., Ito Y., Shiba H., Seki T., Ariga T. (2005). Diallyl trisulfide suppresses the proliferation and induces apoptosis of human colon cancer cells through oxidative modification of beta-tubulin. J. Biol. Chem..

[29] Knowles L.M., Milner J.A. (2000). Diallyl disulfide inhibits p34(cdc2) kinase activity through changes in complex formation and phosphorylation. Carcinogenesis.

[30] Filomeni G., Aquilano K., Rotilio G., Ciriolo M.R. (2003). Reactive oxygen species-dependent c-Jun NH2-terminal kinase/c-Jun signaling cascade mediates neuroblastoma cell death induced by diallyl disulfide. Cancer Res..

[31] Wu C.C., Chung J.G., Tsai S.J., Yang J.H., Sheen L.Y. (2004). Differential effects of allyl sulfides from garlic essential oil on cell cycle regulation in human liver tumor cells. Food Chem. Toxicol..

[32] Yuan J.P., Wang G.H., Ling H., Su Q., Yang Y.H., Song Y., Tang R.J., Liu Y., Huang C. (2004). Diallyl disulfide-induced G2/M arrest of human gastric cancer MGC803 cells involves activation of p38 MAP kinase pathways. World J. Gastroenterol..

[33] Xiao D., Herman-Antosiewicz A., Antosiewicz J., Xiao H., Brisson M., Lazo J.S., Sing S.V. (2005). Diallyl trisulfide-induced G(2)-M phase cell cycle arrest in human prostate cancer cells is caused by reactive oxygen species-dependent destruction and hyperphosphorylation of Cdc 25 C. Oncogene.

[34] Antosiewicz J., Herman-Antosiewicz A., Marynowski S.W., Singh S.V. (2006). c-Jun NH(2)-terminal kinase signaling axis regulates diallyl trisulfide-induced generation of reactive oxygen species and cell cycle arrest in human prostate cancer cells. Cancer Res..

[35] Herman-Antosiewicz A., Stan S.D., Hahm E.-R., Xiao D., Singh S.V. (2007). Activation of a novel ataxia-telangiectasia mutated and Rad3 related/checkpoint kinase 1-dependent prometaphase checkpoint in cancer cells by diallyl trisulfide, a promising cancer chemopreventive constituent of processed garlic. Mol. Cancer Ther..

[36] Hosono T., Hosono-Fukao T., Inada K., Tanaka R., Yamada H., Iitsuka Y., Seki T., Hasegawa I., Ariga T. (2008). Alkenyl group is responsible for the disruption of microtubule network formation in human colon cancer cell line HT-29 cells. Carcinogenesis.

[37] Wu X.J., Hu Y., Lamy E., Mersch-Sundermann V. (2009). Apoptosis induction in human lung adenocarcinoma cells by oil-soluble allyl sulfides: Triggers, pathways, and modulators. Environ. Mol. Mutagen..

[38] Xiao D., Zeng Y., Hahm E.R., Kim Y.A., Ramalingam S., Singh S.V. (2009). Diallyl trisulfide selectively causes Bax- and Bak-mediated apoptosis in human lung cancer cells. Environ. Mol. Mutagen..

[39] Xiao D., Zeng Y., Singh S.V. (2009). Diallyl trisulfide-induced apoptosis in human cancer cells is linked to checkpoint kinase 1-mediated mitotic arrest. Mol. Carcinog..

[40] Herman-Antosiewicz A., Kim Y.A., Kim S.-H., Xiao D., Sing S.V. (2010). Diallyl trisulfide-induced G2/M phase cell cycle arrest in DU145 cells is associated with delayed nuclear translocation of cyclin-dependent kinase 1. Pharm. Res..

[41] Wang Y.B., Qin J., Zheng X.Y., Bai Y., Yang K., Xie L.P. (2010). Diallyl trisulfide induces Bcl-2 and caspase-3-dependent apoptosis via downregulation of Akt phosphorylation in human T24 bladder cancer cells. Phytomedicine.

[42] Wang H.C., Yang J.H., Hsieh S.C., Sheen L.Y. (2010). Allyl sulfides inhibit cell growth of skin cancer cells through induction of DNA damage mediated G2/M arrest and apoptosis. J. Agric. Food Chem..

[43] Borkowska A., Sielicka-Dudzin A., Herman-Antosiewicz A., Halon M., Wozniak M., Antosiewicz J. (2011). P66Shc mediated ferritin degradation—A novel mechanism of ROS formation. Free Radic. Biol. Med..

[44] Chen M., Li B., Zhao X., Zuo H., He X., Li Z., Liu X., Chen L. (2012). Effect of diallyl trisulfide derivatives on the induction of apoptosis in human prostate cancer PC-3 cells. Mol. Cell. Biochem..

[45] Wang H.C., Hsieh S.C., Yang J.H., Lin S.Y., Sheen L.Y. (2012). Diallyl trisulfide induces apoptosis of human basal cell carcinoma cells via endoplasmic reticulum stress and the mitochondrial pathway. Nutr. Cancer.

[46] Pan Y., Lin S., Xing R., Zhu M., Lin B., Cui J., Li W., Gao J., Shen L., Zhao Y. (2016). Epigenetic upregulation of metallothionein 2A by diallyl trisulfide enhances chemosensitivity of human gastric cancer cells to docetaxel through attenuating NF-κB activation. Antioxid. Redox Signal..

[47] Borkowska A., Knap N., Antosiewicz J. (2013). Diallyl trisulfide is more cytotoxic to prostate cancer cells PC-3 than to noncancerous epithelial cell line PNT1A: A possible role of p66Shc signaling axis. Nutr. Cancer.

[48] Sielicka-Dudzin A., Borkowska A., Herman-Antosiewicz A., Wozniak M., Jozwik A., Fedeli D., Antosiewicz J. (2012). Impact of JNK1, JNK2, and ligase Itch on reactive oxygen species formation and survival of prostate cancer cells treated with diallyl trisulfide. Eur. J. Nutr..

[49] Bauer G. (2002). Signaling and proapoptotic functions of transformed cell-derived reactive oxygen species. Prostaglandins leukot. Essent. Fatty Acids.

[50] Shankar S., Chen Q., Ganapathy S., Singh K.P., Srivastava R.K. (2008). Diallyl trisulfide increases the effectiveness of TRAIL and inhibits prostate cancer growth in an orthotopic model: Molecular mechanisms. Mol. Cancer Ther..

[51] Singh S.V., Powolny A.A., Stan S.D., Xiao D., Arlotti J.A., Warin R., Hahm E.R., Marynowski S.W., Bommareddy A., Potter D.M. (2008). Garlic constituent diallyl trisulfide prevents development of poorly differentiated prostate cancer and pulmonary metastasis multiplicity in TRAMP mice. Cancer Res..

[52] Kim S.H., Bommareddy A., Singh S.V. (2011). Garlic constituent diallyl trisulfide suppresses x-linked inhibitor of apoptosis protein in prostate cancer cells in culture and in vivo. Cancer Prev. Res..

[53] Chandra-Kuntal K., Singh S.V. (2010). Diallyl trisulfide inhibits activation of signal transducer and activator of transcription 3 in prostate cancer cells in culture and in vivo. Cancer Prev. Res..

[54] Stan S.D., Singh S.V. (2009). Transcriptional repression and inhibition of nuclear translocation of androgen receptor by diallyl trisulfide in human prostate cancer cells. Clin. Cancer Res..

[55] Lai K.C., Hsu S.C., Yang J.S., Yu C.C., Lein J.C., Chung J.G. (2015). Diallyl trisulfide inhibits migration, invasion and angiogenesis of human colon cancer HT-29 cells and umbilical vein endothelial cells, and suppresses murine xenograft tumour growth. J. Cell. Mol. Med..

[56] Wu P.P., Liu K.C., Huang W.W., Chueh F.S., Ko Y.C., Chiu T.H., Lin J.P., Kuo J.H., Yang J.S., Chung J.G. (2011). Diallyl trisulfide (DATS) inhibits mouse colon tumor in mouse CT-26 cells allograft model in vivo. Phytomedicine.

[57] Na H.K., Kim E.H., Choi M.A., Park J.M., Kim D.H., Surh Y.J. (2012). Diallyl trisulfide induces apoptosis in human breast cancer cells through ROS-mediated activation of JNK and AP-1. Biochem. Pharmacol..

[58] Kim S.H., Kaschula C.H., Priedigkeit N., Lee A.V., Singh S.V. (2016). Forkhead box Q1 is a novel target of breast cancer stem cell inhibition by diallyl trisulfide. J. Biol. Chem..

[59] Liu Y., Zhu P., Wang Y., Wei Z., Tao L., Zhu Z., Sheng X., Wang S., Ruan J., Liu Z. (2015). Antimetastatic therapies of the polysulfide diallyl trisulfide against triple-negative breast cancer (TNBC) via suppressing MMP2/9 by blocking NF-κB and ERK/MAPK signaling pathways. PLoS ONE.

[60] Kim S., Lee H.G., Park S.A., Kundu J.K., Keum Y.S., Cha Y.N., Na H.K., Surh Y.J. (2014). Keap1 cysteine 288 as a potential target for diallyl trisulfide-induced Nrf2 activation. PLoS ONE.

[61] Li W., Tian H., Li L., Li S., Yue W., Chen Z., Qi L., Hu W., Zhu Y., Hao B. (2012). Diallyl trisulfide induces apoptosis and inhibits proliferation of A549 cells in vitro and in vivo. Acta Biochim. Biophys. Sin..

[62] Shrotriya S., Kundu J.K., Na H.K., Surh Y.J. (2010). Diallyl trisulfide inhibits phorbol ester-induced tumor promotion, activation of AP-1, and expression of COX-2 in mouse skin by blocking JNK and Akt signaling. Cancer Res..

[63] Wallace G.C., Haar C.P., Vandergrift W.A., Giglio P., Dixon-Mah Y.N., Varma A.K., Ray S.K., Patel S.J., Banik N.L., Das A. (2013). Multi-targeted DATS prevents tumor progression and promotes apoptosis in ectopic glioblastoma xenografts in SCID mice via HDAC inhibition. J. Neurooncol..

[64] Hung F.M., Shang H.S., Tang N.Y., Lin J.J., Lu K.W., Lin J.P., Ko Y.C., Yu C.C., Wang H.L., Liao J.C. (2015). Effects of diallyl trisulfide on induction of apoptotic death in murine leukemia WEHI-3 cells in vitro and alterations of the immune responses in normal and leukemic mice in vivo. Environ. Toxicol..

[65] Savitsky P.A., Finkel T. (2002). Redox regulation of Cdc25C. J. Biol. Chem..

[66] Malki A., El-Saadani M., Sultan A.S. (2009). Garlic constituent diallyl trisulfide induced apoptosis in MCF-7 human breast cancer cells. Cancer Biol. Ther..

[67] Ma H.B., Huang S., Yin X.R., Zhang Y., Di Z.L. (2014). Apoptotic pathway induced by diallyl trisulfide in pancreatic cancer cells. World J. Gastroenterol..

[68] Li Y., Zhang J., Zhang L., Si M., Yin H., Li J. (2013). Diallyl trisulfide inhibits proliferation, invasion and angiogenesis of osteosarcoma cells by switching on suppressor microRNAs and inactivating of Notch-1 signaling. Carcinogenesis.

[69] Das A., Banik N.L., Ray S.K. (2007). Garlic compounds generate reactive oxygen species leading to activation of stress kinases and cysteine proteases for apoptosis in human glioblastoma T98G and U87MG cells. Cancer.

[70] Kim Y.A., Xiao D., Xiao H., Powolny A.A., Lew K.L., Reilly M.L., Zeng Y., Wang Z., Singh S.V. (2007). Mitochondria-mediated apoptosis by diallyl trisulfide in human prostate cancer cells is associated with generation of reactive oxygen species and regulated by Bax/Bak. Mol. Cancer Ther..

[71] Yu C.S., Huang A.C., Lai K.C., Huang Y.P., Lin M.W., Yang J.S., Chung J.G. (2012). Diallyl trisulfide induces apoptosis in human primary colorectal cancer cells. Oncol. Rep..

[72] Chandra-Kuntal K., Lee J., Singh S.V. (2013). Critical role for reactive oxygen species in apoptosis induction and cell migration inhibition by diallyl trisulfide, a cancer chemopreventive component of garlic. Breast Cancer Res. Treat..

[73] Borkowska A., Sielicka-Dudzin A., Herman-Antosiewicz A., Wozniak M., Fedeli D., Falcioni G., Antosiewicz J. (2012). Diallyl trisulfide-induced prostate cancer cell death is associated with Akt/PKB dephosphorylation mediated by P-p66shc. Eur. J. Nutr..

[74] Torti S.V., Torti F.M. (2013). Iron and cancer: More ore to be mined. Nat. Rev. Cancer.

[75] Chen C., Pung D., Leong V., Hebbar V., Shen G., Nair S., Li W., Kong A.N. (2004). Induction of detoxifying enzymes by garlic organosulfur compounds through transcription factor Nrf2: Effect of chemical structure and stress signals. Free Radic. Biol. Med..

[76] Xiao D., Choi S., Johnson D.E., Vogel V.G., Johnson C.S., Trump D.L., Lee Y.J., Singh S.V. (2004). Diallyl trisulfide-induced apoptosis in human prostate cancer cells involves c-Jun N-terminal kinase and extracellular-signal regulated kinase-mediated phosphorylation of Bcl-2. Oncogene.

[77] Lee B.C., Park B.H., Kim S.Y., Lee Y.J. (2011). Role of Bim in diallyl trisulfide-induced cytotoxicity in human cancer cells. J. Cell. Biochem..

[78] Shin D.Y., Kim G.Y., Hwang H.J., Kim W.J., Choi Y.H. (2014). Diallyl trisulfide-induced apoptosis of bladder cancer cells is caspase-dependent and regulated by PI3K/Akt and JNK pathways. Environ. Toxicol. Pharmacol..

[79] Fridman J.S., Lowe S.W. (2003). Control of apoptosis by p53. Oncogene.

[80] Wan H.F., Yu L.H., Wu J.L., Tu S., Zhu W.F., Zhang X.L., Wan F.S. (2013). Effect of diallyl trisulfide on human ovarian cancer SKOV-3/DDP cell apoptosis. Asian Pac. J. Cancer Prev..

[81] Lan H., Lü Y. (2004). Allitridi induces apoptosis by affecting Bcl-2 expression and caspase-3 activity in human gastric cancer cells. Acta Pharmacol. Sin..

[82] Zhou C., Mao X.P., Guo Q., Zeng F.Q. (2009). Diallyl trisulphide-induced apoptosis in human melanoma cells involves downregulation of Bcl-2 and Bcl-xL expression and activation of caspases. Clin. Exp. Dermatol..

[83] Xiao D., Singh S.V. (2006). Diallyl trisulfide, a constituent of processed garlic, inactivates Akt to trigger mitochondrial translocation of BAD and caspase-mediated apoptosis in human prostate cancer cells. Carcinogenesis.

[84] Sakamoto K., Lawson L.D., Milner J.A. (1997). Allyl sulfides from garlic suppress the in vitro proliferation of human A549 lung tumor cells. Nutr. Cancer.

[85] Ji C., Ren F., Xu M. (2010). Caspase-8 and p38MAPK in DATS-induced apoptosis of human CNE2 cells. Braz. J. Med. Biol. Res..

[86] Lai K.C., Hsu S.C., Kuo C.L., Yang J.S., Ma C.Y., Lu H.F., Tang N.Y., Hsia T.C., Ho H.C., Chung J.G. (2013). Diallyl sulfide, diallyl disulfide, and diallyl trisulfide inhibit migration and invasion in human colon cancer colo 205 cells through the inhibition of matrix metalloproteinase-2, -7, and -9 expressions. Environ. Toxicol..

[87] Iitsuka Y., Tanaka Y., Hosono-Fukao T., Hosono T., Seki T., Ariga T. (2010). Relationship between lipophilicity and inhibitory activity against cancer cell growth of nine kinds of alk(en)yl trisulfides with different side chains. Oncol. Res..

[88] Lai K.C., Kuo C.L., Ho H.C., Yang J.S., Ma C.Y., Lu H.F., Huang H.Y., Chueh F.S., Yu C.C., Chung J.G. (2012). Diallyl sulfide, diallyl disulfide and diallyl trisulfide affect drug resistant gene expression in colo 205 human colon cancer cells in vitro and in vivo. Phytomedicine.

[89] Shin D.Y., Cha H.J., Kim G.Y., Kim W.J., Choi Y.H. (2013). Inhibiting invasion into human bladder carcinoma 5637 cells with diallyl trisulfide by inhibiting matrix metalloproteinase activities and tightening tight junctions. Int. J. Mol. Sci..

[90] Iitaka D., Moodley S., Shimizu H., Bai X.H., Liu M. (2015). PKCδ-iPLA2-PGE2-PPARγ signaling cascade mediates TNF-α induced Claudin 1 expression in human lung carcinoma cells. Cell Signal..

[91] Shang X., Lin X., Alvarez E., Manorek G., Howell S.B. (2012). Tight junction proteins claudin-3 and claudin-4 control tumor growth and metastases. Neoplasia.

[92] Lin X., Shang X., Manorek G., Howell S.B. (2013). Regulation of the epithelial-mesenchymal transition by Claudin-3 and Claudin-4. PLoS ONE.

[93] Mitra S., Lee J.S., Cantrell M., van Den Berg C.L. (2011). c-Jun N-terminal kinase 2 (JNK2) enhances cell migration through epidermal growth factor substrate 8 (EPS8). J. Biol. Chem..

[94] Xiao D., Li M., Herman-Antosiewicz A., Antosiewicz J., Xiao H., Lew K.L., Zeng Y., Marynowski S.W., Singh S.V. (2006). Diallyl trisulfide inhibits angiogenic features of human umbilical vein endothelial cells by causing Akt inactivation and down-regulation of VEGF and VEGF-R2. Nutr. Cancer.

[95] Hahm E.R., Singh S.V. (2014). Diallyl trisulfide inhibits estrogen receptor-α activity in human breast cancer cells. Breast Cancer Res. Treat..

[96] Wang H.C., Pao J., Lin S.Y., Sheen L.Y. (2012). Molecular mechanisms of garlic-derived allyl sulfides in the inhibition of skin cancer progression. Ann. N. Y. Acad. Sci..

[97] Srivastava S.K., Hu X., Xia H., Zaren H.A., Chatterjee M.L., Agarwal R., Singh S.V. (1997). Mechanism of differential efficacy of garlic organosulfides in preventing benzo(a)pyrene-induced cancer in mice. Cancer Lett..

[98] Chang H.S., Ko M., Ishizuka M., Fujita S., Yabuki A., Hossain M.A., Yamato O. (2010). Sodium 2-propenyl thiosulfate derived from garlic induces phase II detoxification enzymes in rat hepatoma H4IIE cells. Nutr. Res..

[99] Stan S.D., Kar S., Stoner G.D., Singh S.V. (2008). Bioactive food components and cancer risk reduction. J. Cell. Biochem..

[100] Kiesel V.A., Stan S.D. (2017). Diallyl trisulfide, a chemopreventive agent from *Allium* vegetables, inhibits alpha-secretases in breast cancer cells. Biochem. Biophys. Res. Commun..

[101] Liang D., Wu H., Wong M.W., Huang D. (2015). Diallyl trisulfide is a fast H2S donor, but diallyl disulfide is a slow one: The reaction pathways and intermediates of glutathione with polysulfides. Org. Lett..

[102] Benavides G.A., Squadrito G.L., Mills R.W., Patel H.D., Isbell T.S., Patel R.P., Darley-Usmar V.M., Doeller J.E., Kraus D.W. (2007). Hydrogen sulfide mediates the vasoactivity of garlic. Proc. Natl. Acad. Sci. USA.

[103] Zhang F., Jin H., Wu L., Shao J., Zhu X., Chen A., Zheng S. (2017). Diallyl trisulfide suppresses oxidative stress-induced activation of hepatic stellate cells through production of hydrogen sulfide. Oxid. Med. Cell. Longev..

[104] Wu D., Si W., Wang M., Lv S., Ji A., Li Y. (2015). Hydrogen sulfide in cancer: Friend or foe?. Nitric Oxide.

[105] Hellmich M.R., Szabo C. (2015). Hydrogen sulfide and cancer. Handb. Exp. Pharmacol..

[106] Liu M., Wu L., Montaut S., Yang G. (2016). Hydrogen sulfide signaling axis as a target for prostate cancer therapeutics. Prostate Cancer.

[107] Jiang X.Y., Zhu X.S., Xu H.Y., Zhao Z.X., Li S.Y., Li S.Z., Cai J.H., Cao J.M. (2017). Diallyl trisulfide suppresses tumor growth through the attenuation of Nrf2/Akt and activation of p38/JNK and potentiates cisplatin efficacy in gastric cancer treatment. Acta Pharmacol. Sin..

[108] You W.C., Brown L.M., Zhang L., Li J.Y., Jin M.L., Chang Y.S., Ma J.L., Pan K.F., Liu W.D., Hu Y. (2006). Randomized double-blind factorial trial of three treatments to reduce the prevalence of precancerous gastric lesions. J. Natl. Cancer Inst..

[109] Gail M.H., You W.C. (2006). A factorial trial including garlic supplements assesses effect in reducing precancerous gastric lesions. J. Nutr..

[110] Ma J.L., Zhang L., Brown L.M., Li J.Y., Shen L., Pan K.F., Liu W.D., Hu Y., Han Z.X., Crystal-Mansour S. (2012). Fifteen-year effects of Helicobacter pylori, garlic, and vitamin treatments on gastric cancer incidence and mortality. J. Natl. Cancer Inst..

[111] Tu H.K., Pan K.F., Zhang Y., Li W.Q., Zhang L., Ma J.L., Li J.Y., You W.C. (2010). Manganese superoxide dismutase polymorphism and risk of gastric lesions, and its effects on chemoprevention in a Chinese population. Cancer Epidemiol. Biomark. Prev..

[112] Ishikawa H., Saeki T., Otani T., Suzuki T., Shimozuma K., Nishino H., Fukuda S., Morimoto K. (2006). Aged garlic extract prevents a decline of NK cell number and activity in patients with advanced cancer. J. Nutr..

[113] Tanaka S., Haruma K., Yoshihara M., Kajiyama G., Kira K., Amagase H., Chayama K. (2006). Aged garlic extract has potential suppressive effect on colorectal adenomas in humans. J. Nutr..

[114] Li H., Li H.Q., Wang Y., Xu H.X., Fan W.T., Wang M.L., Sun P.H., Xie X.Y. (2004). An intervention study to prevent gastric cancer by micro-selenium and large dose of allitridum. Chin. Med. J..

